# Reduction in Urinary Chemokine (C-C Motif) Ligand 2 (CCL2) After Surgery-Induced Weight Loss

**DOI:** 10.1038/s41598-020-57763-8

**Published:** 2020-01-21

**Authors:** Surita Binti Said, Guo Hou Loo, Nik Ritza Kosai, Reynu Rajan, Rozita Mohd, Asrul Abdul Wahab, Shamsul Azhar Shah

**Affiliations:** 1 Department of Surgery, Pusat Perubatan Universiti Kebangsaan Malaysia, Kuala Lumpur, Postcode 56000 Malaysia; 2 Department of Internal Medicine, Pusat Perubatan Universiti Kebangsaan Malaysia, Kuala Lumpur, Postcode 56000 Malaysia; 3 Department of Medical Diagnostic Service, Pusat Perubatan Universiti Kebangsaan Malaysia, Kuala Lumpur, Postcode 56000 Malaysia; 4 Department of Community Health, Pusat Perubatan Universiti Kebangsaan Malaysia, Kuala Lumpur, Postcode 56000 Malaysia

**Keywords:** Obesity, Chronic kidney disease, Diagnostic markers

## Abstract

Kidney dysfunction, a deleterious effect of obesity, is now recognized as a relevant health risk. Chemokine (C-C Motif) Ligand 2 (CCL2) is one of the critical chemokines that play a vital role in the development of obesity-related metabolic disease. We aim to measure the changes in urinary CCL2 in our patients before and after their bariatric procedure and examine the correlation between CCL2 and renal function. A prospective cohort study was conducted at our teaching university hospital. Ethics approval was obtained from our institutional review board. Patients with a BMI of ≥37.5 kg/m^2^ with no history of renal disease were included. They underwent single anastomosis gastric bypass (SAGB), Roux-en-Y gastric bypass (RYGB) or sleeve gastrectomy (SG), all performed via laparoscopic approach. Venous blood and urine samples were obtained preoperatively and six months after surgery. A total of 58 patients were recruited, with SG being performed in 74.1% of patients. At six-months follow-up, median (IQR) body weight reduced from 101.35 kgs (20.25) to 76.95 kg (24.62) *p* < 0.001. The mean (SD) estimated glomerular filtration rate (eGFR) improved from 96.26 ± 14.97 to 108.06 ± 15.00 mL/min/1.73 m^2^, *p* < 0.001. The median (IQR) urinary CCL2 levels reduced from 15.2 pg/ml (10.77) to 4.30 pg/ml (4.27) *p < *0·001. There is a significant correlation between the reduction of BMI and the reduction of urinary CCL2 (r = −0.220, p = 0.048). We also found a significant correlation between the reduction of urinary CCL2 with the reduction of urine ACR (r = −0.240, p = 0.035). Urinary CCL2 is a promising biomarker that can be used to assess improvement in renal function in obese patients after bariatric surgery.

## Introduction

The prevalence of obesity is rapidly growing worldwide and is a major causative factor for many metabolic disorders. In 2005, there were about 1.6 billion overweight people aged ≥15 years, and among them, at least 400 million obese adults^1^. Likewise, the prevalence of overweight and obesity has increased many folds in the past few decades in most Asian countries^2^. In Malaysia, the prevalence of obesity and overweight has drastically increased from 4.4% in 1996 to 17.7% in 2015^3^.

Kidney dysfunction is one of the deleterious effects of obesity. Changes in renal function induced by obesity include albuminuria, hyperfiltration, as well as reduced glomerular filtration rate^4^. Obesity-related glomerulopathy (ORG), a distinct histologic pattern of glomerulomegaly and thickened glomerular basement membrane, has also been identified^5^. The progression of ORG may eventually lead to established chronic kidney disease. ORG induces renal injury via adipokines, renin-angiotensin-aldosterone system, lipids, sympathetic nervous system, apoptosis, and oxidative stress^6^.

Recognition of obesity as a state of chronic low-grade inflammation is one of the significant recent developments in obesity research^7^. CCL2 is one of the critical chemokines that play a vital role in the development and progression of obesity-related metabolic disease via the inflammatory pathway^8,9^. Based on previous studies, the measured level of serum CCL2 is significantly higher in obese patients when compared to lean controls^8,9^ and this level was reduced following bariatric surgery^10^. CCL2 is strongly associated with renal disease in both obesity and diabetic nephropathy^11^. An association of urinary CCL2 level with obesity-related co-morbidities was also found^12^.

Our study aims to measure the changes in urinary CCL2 in our patients before and after their weight-loss surgery. We also analyzed their renal function and its possible correlation with urinary CCL2. We postulated that reduction in urinary CCL2 level after bariatric surgery would coincide with an improvement in renal function as well.

## Results

A total of 58 patients were recruited, in which 68.9% (n = 40) were women. The mean (SD) age was 38.79 ± 8.40 years. The majority of patients were of Malay ethnicity (86.2%). The median (IQR) body weight before surgery was 101.35 kgs (20.25) with a median (IQR) BMI of 45.0 kg/m^2^ (7.655). The mean (SD) eGFR and median(IQR) urine ACR before surgery was 96.26 (14.97) and 2.5 (3.7), respectively. About 24% of our patients had a diabetic range of HbA1c level (≥6.5%) before surgery. The median(IQR) HbA1c level was 5.7% (0.95). Baseline characteristic of patients with anthropometric measurement prior to bariatric surgery are summarised in Table 1.

**Table 1:** Baseline characteristic of study participants before surgery.

Variables (n = 58)	
Mean Age (SD) in years	38.79 ± 8.4
**Gender, no (%)**	
Male	18 (31)
Female	40 (69)
**Ethnicity, no (%)**	
Malay	50 (86.2)
Chinese	2 (3.4)
Indian	6 (10.3)
Mean (SD) eGFR in mL/min/1.73 m²	96.26 (14.97)
Male	102.03 (17.70)
Female	93.66 (13.00)
Weight in kg, median (IQR)	101.35 (20.25)
Male	99.61 (15.85)
Female	102.40 (20.19)
BMI in kg/m², median (IQR)	45.00 (7.65)
Male	47.45 (7.68)
Female	44.14 (5.82)
Serum creatinine in mmol/L, median (IQR)	68.45 (12.78)
Male	75.20 (18.83)
Female	65.70 (9.58)
eGFR in mL/min/1.73 m², mean (SD)	96.26 (14.97)
Male	102.03 (17.70)
Female	93.66 (13.00)
Urine ACR, median (IQR)	25.00 (37.0)
Male	32.00 (42.75)
Female	19.00 (38.00)
Microalbuminuria, n (%)	25 (43.1)
Macroalbuminuria, n (%)	1 (1.72)
HbA1c in %, median (IQR)	5.70 (0.95)
Male	5.75 (1.25)
Female	5.70 (0.95)
Urinary CCL2 in pg/ml, median (IQR)	15.19 (10.77)
Male	14.72 (13.54)
Female	15.25 (10.12)
**Type of Operation, no (%)**	
SG	43 (74.1)
RYGB	6 (10.3)
SAGB	9 (15.5)

eGFR (estimated Glomerular Filtration Rate), HbA1C (Glycated haemoglobin A1c), SG (sleeve gastrectomy), RYGB (Roux-en-Y gastric bypass), SAGB (single anastomosis gastric bypass), IQR (interquartile range), SD (standard deviation), urine ACR (urine albumin-creatinine ratio), urinary CCL2 (chemokine C-C motif ligand 2).

At six months follow-up, all baseline parameters showed significant improvement. The median (IQR) body weight significantly reduced from 101.35 kgs (20.25) to 76.95 kg (24.62) *p* < 0.001 and the median (IQR) BMI reduced from 45.00 kg/m^2^ (7.655) to 34.50 kg/m^2^ (6.60), *p* < 0.001. The mean percentage (SD) of weight difference was 25.00 ± 5.38%. The median (IQR) HbA1c level reduced from 5.7% (0.95) to 5.3% (0.43) *p* < 0.001. Renal parameters such as urine ACR, serum creatinine, and eGFR all showed significant improvement as well. Of note, a significant reduction of urinary CCL2 is demonstrated in our patients, with 67.2% of patients showing a reduction of ≥50%. The median (IQR) urinary CCL2 reduced from 15.2 (10.77) to 4.30 (4.27) *p* < 0.001 and occurs uniform across both genders.

At baseline, 25 patients (43.1%) fell into the category of microalbuminuria (urine ACR between 30 to 300), and only one patient had macroalbuminuria (urine ACR > 300.0). At six- months follow-up, a significant reduction of urine ACR was evident, p < 0.001, with the number of patients with microalbuminuria reduced to only three (5.17%). The baseline median (IQR) of urinary CCL2 for patients without microalbuminuria was 16.16 (15.73) and 15.21(8.50) for patients with microalbuminuria. Using the Mann Whitney U test, there was no significant correlation between both groups (z = −0.088, p = 0.93). Interestingly, the sole patient with macroalbuminuria did not have the highest level of urinary CCL2. The change in baseline parameters before and after surgery are illustrated in Table 2.

**Table 2:** Difference in baseline parameters before and six months after bariatric surgery.

Variables	Pre-Operation	Post-Operation	*p-value*
Weight in kgs, median (IQR)	101.35 (20.25)	76.95 (24.62)	<0.001
Male	99.61 (15.85)	67.70 (19.69)	<0.001
Female	102.40 (20.19)	79.27 (26.47)	<0.001
BMI in kg/m², median (IQR)	45.00 (7.65)	34.50 (6.6)	<0.001
Male	47.45 (7.68)	31.05 (7.65)	<0.001
Female	44.14 (5.82)	34.65 (6.13)	<0.001
Serum Creatinine in mmol/L, median (IQR)	68.45 (12.77)	61.25 (13.65)	<0.001
Male	75.20 (18.82)	72.40 (9.3)	<0.001
Female	65.70 (9.57)	59.40 (8.42)	<0.001
eGFR in mL/min/1.73 m², mean (SD)	96.26 (14.97)	108.06 (15.00)	<0.001
Male	102.03 (17.70)	112.83 (15.86)	<0.001
Female	93.66 (13.00)	105.92 (14.29)	<0.001
Urine ACR, median (IQR)	25.00 (37.00)	5.00 (7.25)	<0.001
Male	32.00 (42.75)	6.50 (11.00)	<0.001
Female	19.00 (38.00)	5.00 (5.75)	<0.001
Microalbuminuria, n (%)	25 (43.10)	3 (5.17)	
Macroalbuminuria, n (%)	1 (1.72)	0 (0)	
HbA1c in %, median (IQR)	5.70 (0.95)	5.30 (0.43)	<0.001
Male	5.75 (1.25)	5.20 (0.57)	0.001
Female	5.70 (0.95)	5.30 (0.40)	<0.001
CCL2 in pg/ml, median (IQR)	15.19 (10.77)	4.30 (4.27)	<0.001
Male	14.72 (13.54)	4.42 (4.17)	<0.001
Female	15.25 (10.12)	4.20 (4.28)	<0.001
CCL2 reduction 50% and more compared to baseline, n (%)		39 (67.2)	
Male		12 (66.7)	
Female		27 (67.5)	

BMI (body mass index), eGFR (estimated Glomerular Filtration Rate), HbA1C (Glycated haemoglobin A1c), SG (sleeve gastrectomy), RYGB (Roux-en-Y gastric bypass), SAGB (single anastomosis gastric bypass), IQR (interquartile range), SD (standard deviation), urine ACR (urine albumin-creatinine ratio), urinary CCL2 (chemokine C-C motif ligand 2).

There was no significant difference in the baseline and renal function parameters between SG, SAGB, and RYGB. SG was performed for the majority of our patients (74.1%). The changes in baseline and renal parameters six months after surgery are summarised in Table 2.

Overall, there is a significant correlation between the reduction of BMI and the reduction of urinary CCL2 (r = −0.220, p = 0.048). There is also a significant correlation between the reduction of urinary CCL2 with the reduction of urine ACR (r = −0.240, p = 0.035). However, there is no significant correlation between the reduction of urinary CCL2 with the difference in eGFR or serum creatinine before and after surgery. There was no significant difference in the reduction of urinary CCL2 seen between SG, RYGB and SAGB as well.

There is a significant difference in the reduction in BMI (r = 0.360, p = 0.006) and weight difference (r = 0.403, p = 0.002) after surgery across genders. However, we could not elicit any statistically significant correlation between gender and the reduction in urinary CCL2 (r = −0.074, p = 0.582), the reduction of urine ACR (r = 0.192, p = 0.149) and the difference in creatinine (r = 0.01, p = 0.941) six months after surgery.

## Discussion

This study demonstrates a significant reduction of urinary CCL2 after bariatric surgery. CCL2 is an inflammatory cytokine that is involved in many inflammatory activities in the human body. This biomarker is one of the most frequently studied for various inflammatory-induced diseases, including obesity-related kidney disease^11,13^. High levels of serum CCL2 in obese patient results from high expression and production of this chemokine from excessive subcutaneous and visceral adipose tissue among obese patients^8^. A high level of urinary CCL2 was present in our patients, and this level reduced significantly following bariatric surgery. Surgically-induced weight loss may ameliorate renal inflammatory cascade and improve ORG^12^.

Urinary CCL2 has been proposed as a marker of renal injury following the evidence showing a positive correlation between urinary MCP-1 with albuminuria, mesangial proliferation, and interstitial fibrosis^13^. Renal tubular epithelial cells express CCL2 in response to a variety of pro-inflammatory stimuli^14^. This local production of CCL2 in the kidney will lead to higher excretion of this cytokine in the urine. A reduction in excessive adipose tissue following bariatric surgery will cause a decreased CCL2 production, which subsequently eliminates the stimulus for local production of CCL2 from the kidneys^12^.

Our study also demonstrates a significant correlation between urinary CCL2 with urine ACR, a marker of renal damage. CCL2 produced in the renal tubular cells is released into the urine in proportion to the degree of renal damage^14,15^. This CCL2 increases inflammatory molecules on mesangial and tubular cells and induces tubule-intestinal injury via recruitment and activation of macrophage^16^. Persistent stimulation will ultimately lead to *in situ* renal fibrosis and, subsequently, renal damage^14,15^ Thus, urinary CCL2 levels following bariatric surgery may be used as a surrogate marker to the ongoing renal inflammatory process.

Our study has several limitations. We did not include patients with established kidney disease as bariatric surgery in patients with advanced kidney disease carries a higher complication rate^17^. However, a systematic review concluded that bariatric surgery might prevent a decline in renal function by improving glomerular hyperfiltration and reducing albuminuria^18^. More studies are needed to elucidate whether progression to end-stage renal disease can be halted with bariatric surgery.

We also failed to show a significant correlation between an improvement in eGFR with a reduction in urinary CCL2, in contrast to a previous study^12^. It is possible that calculating eGFR using a creatinine-based formula may lead to an overestimation of renal function. A majority of our patients (63.8%) have an eGFR ≥ 90 mL/min/1.73 m^2^ at the beginning of the study, and this increased to 91.4% six months after surgery. The observed decrease in serum creatinine levels after bariatric surgery might reflect the inevitable decrease in lean body mass rather than an actual improvement in renal function^19^.

Of note, cystatin C is an endogenous substance produced continuously and freely filtered in the glomerulus without tubular secretion or reabsorption. Its excretion in the kidney is not affected by body mass or weight loss^20^. The use of cystatin C to measure the glomerular filtration rate may give a more accurate reading. However, studies have shown that cystatin C has a direct relationship with fat mass, and a higher BMI seems to be associated with a higher cystatin C^21,22^. Hence, the use of creatinine or cystatin C based formula both poses their limitations.

Urinary CCL2 as a marker of renal injury in the obese population, has mostly been studied in the western population. To date, only one paper had studied this inflammatory marker and its relation to renal injury in the obese Asian population^10^, and it only included patients of the male gender. A more extensive prospective study evaluating the use of urinary CCL2 to assess improvements in renal function in obese Asian patients with established renal disease is needed.

## Conclusion

Urinary CCL2 is a promising biomarker that can be used to assess improvement in renal function in the obese population after bariatric surgery, as creatinine-based formula often leads to an overestimation of renal function. A reduction in urinary CCL2 after bariatric surgery correlates well with urine ACR and the reduction of BMI, and this reduction is also uniform across genders. In the obese population with early-stage renal disease, bariatric surgery may be beneficial in ameliorating the existing renal inflammation and prevent a decline in renal function.

## Methods

This is a prospective cohort study assessing the change in urinary CCL2 levels before and after bariatric surgery, conducted at a teaching university hospital from June 2016 to June 2018. The study was conducted following the principles of the Declaration of Helsinki. Ethics approval was obtained before the start of this study from our institutional review board, Universiti Kebangsaan Malaysia Research Ethics Committee (reference number: FF-2017-035). This study was also registered with the Thai Clinical Trials Registry with the number TCTR20190825002. Written informed consent was taken prior to study commencement. All data collected were kept confidential, and patients were allowed to refuse participation in the study. Data presented do not identify individuals.

Purposive sampling method was employed. We included patients of both genders aged 18–65 years, with a BMI of ≥37.5 kg/m^2^ with no history of renal disease who are planned for bariatric surgery. Patients who received steroid therapy within six months prior to the surgery, patients with malignancy, patients who are pregnant, and patients with stage 3 A kidney disease and beyond (defined as an eGFR < 60 mL/min/1.73 m^2^) were excluded.

A multidisciplinary team performed a thorough assessment, and the final bariatric procedure to be performed is decided, taking into consideration the patient’s choice as well. These procedures include SAGB, RYGB, and SG, all performed via laparoscopic approach by a consultant upper gastrointestinal surgeon with more than five years’ experience. Venous blood and midstream urine samples were taken preoperatively and six months after the surgery. Midstream urine was collected into sterile containers in the morning. The biochemical evaluation was performed for serum creatinine and HbA1c, urinary CCL2, and urine ACR. Serum creatinine and HbA1c were measured using standard laboratory methods.

EGFR was calculated using the MDRD 4 variable formula^23^. An abnormal glomerular filtration rate is defined as eGFR < 90 mL/min per 1.73 m^2^ based on the National Kidney Foundation Kidney Disease Outcomes Quality Initiative (KDOQI)^24^. All urine samples were centrifuged at 1600g for 10 min at 4 °C, and the supernatant was stored at −80 °C until further analysis. The reading of urine albumin was expressed as urine albumin-creatinine ratio (ACR)^17^.

The level of urinary CCL2 was measured using specific enzyme-linked immunoassay (ELISA) kits (Minneapolis, Minn, USA, R&D Systems). Dilution of urine samples was performed according to the manufacturer’s instructions. Samples were then incubated for two hours at room temperature. After washing the plates, a conjugate was added to erase any unbound substances and was left alone for an hour. A substrate solution was then added after washing the plates. Stop solution was added after incubation for 20 min at room temperature. The absorbance was then read at 450 nm with the correction wavelength set at 540 nm. All samples were assayed in duplicate. Urine CCL2 levels were expressed as pictogram per ml (pg/ml).

The study planned as a continuous response variable based on a previous study^25^. The difference in the response is normally distributed with a standard deviation of 25. If true difference in the mean response is 13, we will need 58 subjects to be able to reject the null hypothesis that this response difference is zero with probability (power) of 0.8, and confidence interval of 95%. Type I error probability associated with this test of this null hypothesis is 0.05^26^.

Variables with normal distribution were expressed as mean ± SD, whereas non-parametric variables were expressed as median (IQR). The significance of differences before and after bariatric surgery was evaluated with a paired *t-test* for parametric data and the Mann Whitney U test for non-parametric data. For comparisons among the LAGB, LRYGB, and LSG groups, the Kruskal Wallis one-way test was used. Correlation between variables was performed using the Pearson Bivariate test. All statistical analyses were performed using SPSS (version 24.0; SPSS, Chicago, IL). Statistical significance was set at *p* < 0.05.
